# Clinical application of ozone hydrotherapy combined with scalp complex acid treatment for moderate to severe scalp seborrheic dermatitis

**DOI:** 10.3389/fmed.2026.1791653

**Published:** 2026-04-16

**Authors:** Fangling Wang, Jun Chen, Qianqian Xu, Yiying Ma, Xiaoxia Shi, Sui Liu

**Affiliations:** Department of Dermatology, Suizhou Hospital, Hubei University of Medicine, Suizhou, Hubei, China

**Keywords:** inflammatory markers, ozone hydrotherapy, scalp complex acid, scalp seborrheic dermatitis, skin

## Abstract

**Introduction:**

This study evaluates the effectiveness of combining ozone hydrotherapy with scalp complex acid in treating scalp seborrheic dermatitis (SSD).

**Methods:**

A total of 96 patients with moderate to severe SSD were randomly assigned to either the Control group (treated with scalp complex acid) or the Observation group (treated with a combination of ozone hydrotherapy and scalp complex acid). The treatment period lasted 4 weeks. SSD severity was evaluated using a 16-point composite score that assessed erythema, scaling, and pruritus. Total skin damage score was rated on a 4-point scale. Scalp sebum levels were measured using a Sebumeter SM 810, and serum levels of IL-1β and TNF-α were quantified using enzyme-linked immunosorbent assay.

**Results:**

After 4 weeks, the Observation group showed significantly greater improvement in erythema, scaling, pruritus, sebum production, and lesion area compared to the Control group. The complete lesion clearance rate was significantly higher in the Observation group (35.6%) than in the Control group (11.6%, *p* = 0.009). Additionally, the Observation group experienced more substantial reductions in skin damage score (*p* < 0.001), Adhesive Scales Flaking Score (ASFS; *p* < 0.001), and scalp sebum content (*p* = 0.001). Serum levels of IL-1β and TNF-α were also significantly lower in the Observation group, with IL-1β reduced to 59.20 ± 16.19 pg/mL (*p* = 0.006) and TNF-α reduced to 68.33 ± 18.95 pg/mL (*p* < 0.001).

**Discussion:**

The combination of ozone hydrotherapy and scalp complex acid showed enhanced therapeutic benefits for SSD, leading to improved clinical outcomes and a reduction in systemic inflammation.

## Introduction

Scalp seborrheic dermatitis (SSD) is a chronic, relapsing inflammatory skin disorder that primarily affects the scalp and other seborrheic areas, such as the face and trunk (1, 2). Clinically, SSD is characterized by erythema, greasy scaling, and pruritus, and may be accompanied by folliculitis or hair loss in severe cases, leading to a substantial decline in quality of life (3, 4). Epidemiological data indicate that SSD affects up to 11.6% of the general population, with incidence peaking during adolescence and early adulthood (5).

The pathogenesis of SSD is driven by a complex interplay among various biological systems: (1) sebum overproduction—hyperactive sebaceous glands provide a lipid-rich substrate conducive to the colonization of *Malassezia* spp. (6, 7); (2) microbial dysbiosis—lipase-producing species such as *M. restricta* and *M. globosa* hydrolyze triglycerides into free fatty acids, which stimulate keratinocytes to release proinflammatory cytokines including IL-17 and TNF-α (8, 9); (3) immune dysregulation-Th1/Th2 imbalance and complement system activation amplify local inflammatory cascades (2, 10); (4) epidermal barrier disruption—impaired stratum corneum integrity increases transepidermal water loss and facilitates penetration of external irritants (10, 11). These processes collectively contribute to a vicious cycle of sebum imbalance, microbial overgrowth, immune activation, and barrier dysfunction.

Current first-line therapies for SSD include antifungal agents (e.g., ketoconazole, selenium sulfide), topical corticosteroids, and keratolytics such as salicylic acid (12, 13). While these treatments offer short-term symptomatic relief, their long-term efficacy is limited. Prolonged use of antifungals may promote *Malassezia* resistance (14), whereas topical corticosteroids are associated with adverse effects including skin atrophy and telangiectasia (15). Moreover, conventional therapies provide minimal benefit in restoring epidermal barrier function and are often associated with high relapse rates following treatment cessation (2, 3). Importantly, their single-target mechanisms fail to address the multifactorial pathogenesis of SSD (7, 12).

Ozone, a highly reactive oxygen species, possesses a broad spectrum of therapeutic effects relevant to inflammatory skin disorders. First, it exhibits potent antimicrobial activity by disrupting microbial membrane integrity, thereby exerting broad-spectrum efficacy against pathogens, such as *Malassezia* spp. and *Staphylococcus aureus* (16). Second, ozone possesses anti-inflammatory properties, suppressing proinflammatory cytokines including TNF-α and IL-6 through inhibition of the NF-κB signaling pathway (16). Third, it modulates immune responses by enhancing endogenous antioxidant systems, such as glutathione peroxidase, thereby mitigating oxidative stress-induced tissue damage (17, 18). Fourth, ozone improves local microcirculation and oxygenation, promoting epidermal regeneration and repair of the skin barrier (18). Preclinical studies have shown that ozone water, at concentrations ranging from 3 to 11 mg/L, provides antimicrobial and anti-inflammatory effects without disrupting skin barrier integrity in animal models (19).

Scalp complex acid formulations, comprising agents such as supramolecular salicylic acid and zinc pyrithione, provide complementary therapeutic mechanisms. Salicylic acid promotes keratolysis and facilitates desquamation, thereby reducing excessive scale formation (12, 20). Zinc pyrithione exhibits antifungal properties through inhibition of *Malassezia* biofilm formation (4, 20). In addition, the acidic pH of these formulations contributes to the regulation of sebum secretion and supports the restoration of physiological skin surface acidity (13, 21). Given the multi-targeted biological effects of ozone hydrotherapy, its combination with scalp complex acid is postulated to produce synergistic benefits across antimicrobial, anti-inflammatory, and barrier-repair pathways.

Building on this rationale, we hypothesized that the combination of ozone hydrotherapy and scalp complex acid could improve SSD by reducing Malassezia colonization, inhibiting key proinflammatory mediators, and enhancing epidermal barrier function.

## Material and methods

### Participants

A total of 118 participants were enrolled at Suizhou Hospital between September 2022 and September 2024. The study was approved by Suizhou Hospital, and all the participants provided informed written consent. After applying the inclusion and exclusion criteria, 96 patients with moderate to severe SSD were randomly assigned to either the Control group (treated with scalp complex acid) or the Observation group (treated with a combination of ozone hydrotherapy and scalp complex acid). This study was a randomized controlled clinical trial using a simple randomization design.

### Study randomization and blinding procedures

This was a randomized controlled trial with simple randomization using an SPSS-generated random-number table. An independent statistician generated 96 random numbers in SPSS. The statistician was not involved in recruitment, treatment, or outcome assessment. The random numbers were ranked in ascending order. Participants with odd sequence numbers were allocated to the Control group, and those with even sequence numbers to the Observation group. The allocation sequence was sealed and kept by the statistician. Unblinding was performed only after all participants completed the 4-week treatment and all outcome data were collected.

Participants were assigned random numbers sequentially at enrollment, and treatment was administered according to the corresponding allocation. Participant blinding was not feasible because the ozone hydrotherapy procedure was different from scalp complex acid treatment. Outcome assessment was single-blinded. Two dermatologists, who were not involved in enrollment or treatment, evaluated efficacy based on clinical symptoms and laboratory results. They were blinded to group assignment.

### Inclusion criteria

Patients were eligible if they met the diagnostic criteria for scalp seborrheic dermatitis outlined in the *Chinese Clinical Dermatology* (2nd ed., 2017), were aged ≥18 years, had not received relevant treatment within the past 4 weeks, and provided informed consent.

### Exclusion criteria

Patients were excluded if they had a known allergy to complex acids; coexisting scalp conditions such as psoriasis or eczema; were pregnant, breastfeeding, or planning to conceive; or had severe underlying disorders involving the cardiovascular, cerebrovascular, hepatic, renal, or hematopoietic systems, or a documented history of psychiatric illness.

### Withdrawal criteria

Participants were excluded from the study if they demonstrated non-compliance with the prescribed treatment regimen, used unauthorized topical agents, voluntarily withdrew due to poor adherence, or experienced serious complications or adverse events during the treatment period.

All withdrawals were due to participant-related factors, including loss to follow-up, voluntary discontinuation, or failure to complete the prescribed ozone hydrotherapy. No withdrawals were related to treatment-associated adverse events, such as scalp irritation, allergy, or pain. Because these participants did not complete the 4-week intervention and lacked post-treatment outcome data, they were excluded from the primary analysis.

### Treatment

Patients in the Control group received topical application of scalp complex acid (Zhejiang Ximeien) once weekly via scalp peeling. In the Observation group, patients received the same compound acid treatment combined with ozone hydrotherapy, administered twice weekly through ozone hydrotherapy using the HZ-2601B ozone hydrotherapy device (Hunan Haizhi). Participants in both groups received a 4-week treatment course.

The main ingredient concentrations of the scalp complex acid include 2% salicylic acid, 4% fruit acid, and 1.5% azelaic acid.

The ozone hydrotherapy device produced ozonated water at a concentration of 5 mg/L, at a temperature of approximately 37 °C. It was applied as a rinse for 20 min per session, with a water flow rate of 5 L/min. During the treatment, attention should be paid to potential adverse reactions, which include: chest tightness, throat irritation, skin redness, skin itching, and gastrointestinal discomfort. Clinical adverse reactions were rare; the most common was skin redness, which typically resolved on its own after treatment.

### Assessment of disease severity

The severity of seborrheic dermatitis was evaluated using a composite 16-point scoring system based on three clinical parameters:

1. Erythema area score: the extent of the largest erythematous lesion was scored as follows: 0 = no erythema; 1 = erythema area ≤ 2 cm^2^; 2 = 2 cm^2^ <erythema area <5 cm^2^; 3 = erythema area ≥5 cm^2^.
2. Scaliness score: the severity of scalp scaling was assessed using the Adhesive Scales Flaking Score (ASFS), a 10-point grading scale: 0 = no scaling; 1–2 = fine, powdery grayish-white flakes; 3–4 = small to medium-sized flakes; 5–6 = large, thin flakes loosely attached to the skin; 7–8 = large adherent flakes; 9–10 = thick white-to-yellow flakes firmly attached to the skin.
3. Pruritus score: the severity of pruritus was assessed based on its frequency and impact on sleep: 0 = no pruritus; 1 = occasional itching; 2 = frequent itching without sleep disturbance; 3 = frequent itching with sleep disturbance.
4. The total score, ranging from 0 to 16, was calculated by summing the scores of erythema, scaling, and pruritus. Disease severity was categorized as mild (0–5 points), moderate (6–9 points), or severe (10–16 points).

### Total skin damage scoring

Clinical scoring of total skin damage was conducted with reference to relevant literature and following the *Guidelines for Clinical Research of New Chinese Medicines* (2002 edition). Symptom and sign severity was assessed using a 4-point scale (0–3) for each parameter. Baseline scores were recorded at enrollment, and post-treatment scores were obtained at the end of the intervention. The total score was calculated by summing individual symptom scores.

### ASFS scoring

The Adherent Scalp Flaking Score (ASFS) is a standardized semiquantitative tool used to assess the severity of scalp scaling. The scalp is divided into eight regions: left and right frontal, parietal (vertex), occipital, and nuchal areas. Each region is scored from 0 to 10 based on the size, thickness, and adherence of flakes. A score of 0 indicates no visible scaling; 1–2 denotes fine, powdery loose flakes (minimal); 3–4 indicates small to moderate flakes (mild); 5–6 reflects large, thin loosely attached flakes (moderate); 7–8 represents large, firmly adherent scales (marked); and 9–10 corresponds to thick, white-to-yellow plaques densely affixed to the scalp (severe). The total score ranges from 0 to 80, with higher scores indicating greater severity.

### Measurement of scalp sebum

Scalp sebum levels were assessed under controlled laboratory conditions (temperature: 20 °C; relative humidity: 40%). Participants were instructed to avoid strenuous physical activity and visible sweating for at least 3 h before measurement, and to remain seated at rest for 20 min before testing. Sebum levels on the frontal, vertex, and occipital regions of the scalp were measured using a Sebumeter SM 810 (Courage+Khazaka electronic GmbH, Germany). The average value from the three sites was recorded as the final scalp sebum level.

### Measurement of serum inflammatory markers

Fasting peripheral blood samples were collected at two time points: baseline (before treatment) and post-treatment. Serum was isolated by centrifugation at 3,000 rpm for 10 min at 4 °C and stored at −80 °C until analysis. The concentrations of IL-1β (ab214025, Abcam, MA, USA) and TNF-α (ab181421, Abcam) in serum were quantified using commercially available enzyme-linked immunosorbent assay kits, following the manufacturer's protocols. All samples were measured in duplicate, and absorbance was read at 450 nm using a microplate reader. The average concentration of each cytokine was calculated and used for further analysis.

### Evaluation of clinical efficacy

The primary endpoint was the overall clinical response rate at week 4. Secondary endpoints were the total skin damage score, ASFS, scalp sebum level, and serum IL-1β and TNF-α levels.

Clinical efficacy was evaluated based on the *Standards for the Diagnosis and Therapeutic Effect of TCM Diseases and Syndromes* (2017 edition), using changes in lesion severity after 4 weeks of treatment. Outcomes were classified as follows: recovery (complete resolution; efficacy index ≥95%), markedly effective (substantial improvement; 70%−94%), effective (moderate improvement; 30%−69%), and ineffective (minimal or no improvement; <30%). The overall response rate was calculated as: (recovery + markedly effective + effective cases) / total cases × 100%.

### Sample size calculation

Sample size was calculated in PASS 15.0 (NCSS LLC, Kaysville, Utah) based on a two-sample comparison of proportions for the primary endpoint (overall response rate). Based on pilot data and previous clinical studies, the anticipated response rates were 70.0% in the Control group and 90.0% in the Observation group. With a two-sided α of 0.05 and 80% power, the minimum required sample size was 39 participants per group. Allowing for a pre-specified 10% dropout rate, at least 43 participants per group were needed. Accordingly, 96 participants were randomized, exceeding the minimum required sample size.

### Statistical analysis

Categorical variables are presented as *n* (%). Age and disease duration showed a non-normal distribution and are presented as median (IQR); between-group comparisons were performed using the Mann–Whitney *U* test. Pre-post comparisons were analyzed using the Wilcoxon signed-rank test. Total skin damage score, ASFS score, scalp sebum content, and serum IL-1β and TNF-α levels were normally distributed based on the Shapiro–Wilk test and are presented as mean ± standard deviation (SD). Group differences and pre–post interaction effects were analyzed using two-way ANOVA. A *p*-value of <0.05 was considered statistically significant. Regarding missing data, all withdrawals were due to loss to follow-up or voluntary discontinuation, and no efficacy or laboratory data were missing; therefore, no imputation was performed.

## Results

### CONSORT flow diagram of participant enrollment and randomization

**Figure 1:**
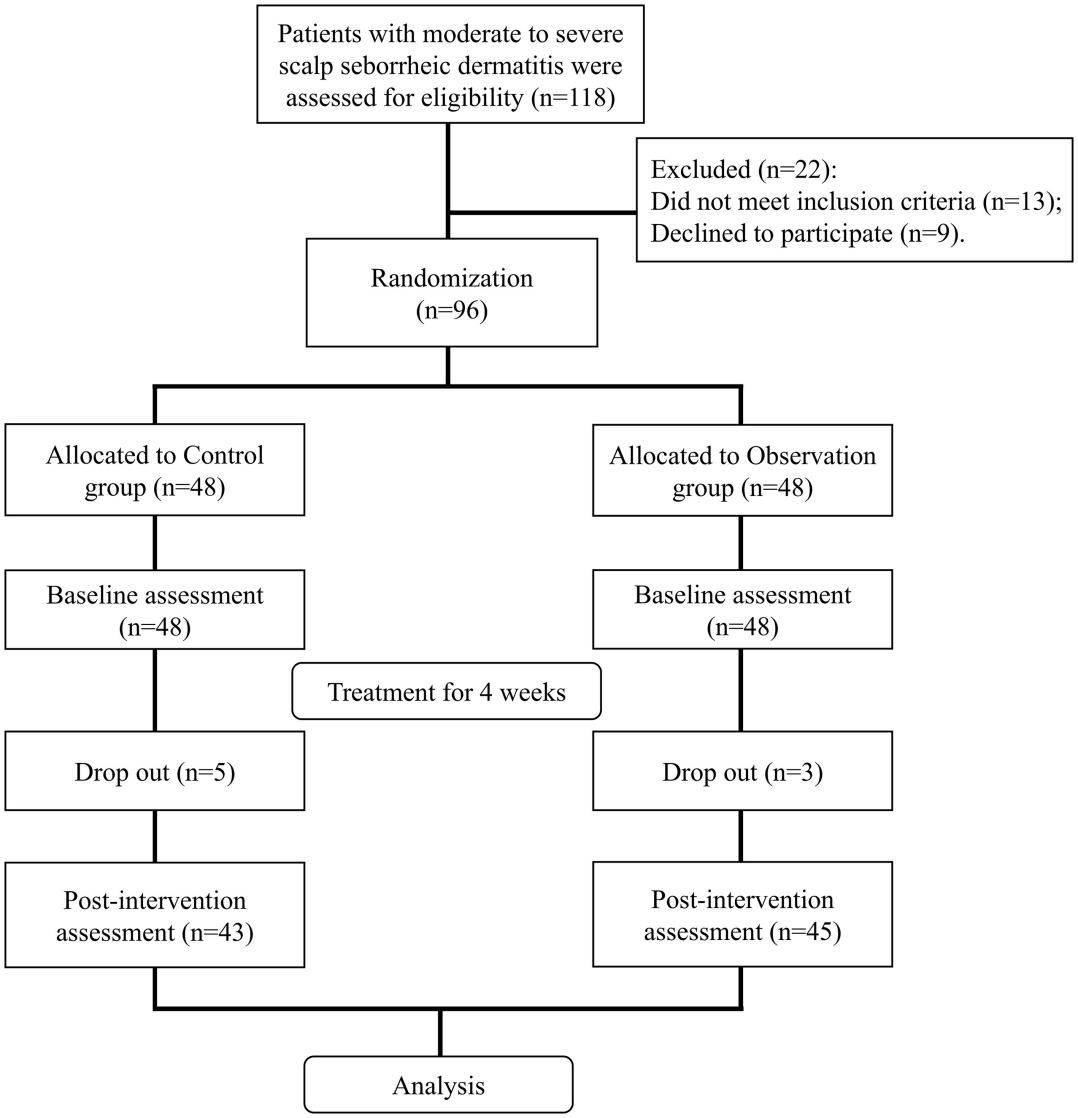
CONSORT flow diagram.

As shown in Figure 1, a total of 118 patients with moderate-to-severe SSD were assessed for eligibility. Among them, 22 were excluded from enrollment: 13 due to failure to meet the inclusion criteria and nine owing to refusal to participate. The remaining 96 participants were randomly assigned to either the Control group (*n* = 48) or the Observation group (*n* = 48). The Control group received 4 weeks of treatment with scalp complex acid, while the Observation group underwent a combined treatment of ozone hydrotherapy and scalp complex acid for 4 weeks. During the treatment period, five participants in the Control group and three in the Observation group withdrew, resulting in final analysis populations of 43 and 45, respectively.

### Demographic and clinical characteristics of patients with moderate to severe scalp seborrheic dermatitis

Baseline demographic and clinical characteristics were comparable between the two groups, as detailed in Table 1. No statistically significant differences were observed in age, disease duration, or gender distribution. The median age was 28 years (IQR: 24.5–35) in the Control group and 28 years (IQR: 24–34.75) in the Observation group (*p* = 0.862). The median duration of disease was 3.5 years (IQR: 1.75–6.5) and 3.75 years (IQR: 2–7) in the Control and Observation groups, respectively (*p* = 0.336). Gender distribution was also similar, with males comprising 41.9% of the Control group and 46.7% of the Observation group (*p* = 0.674).

**Table 1:** Demographic and clinical characteristics of patients with moderate to severe scalp seborrheic dermatitis.

Characteristics	Study group		*p*-value
	Control (*n* = 43)	Observation (*n* = 45)	
Age (years)	28 (24.5, 35)	28 (24, 34.75)	0.862
Course of disease (years)	3.5 (1.75, 6.5)	3.75 (2, 7)	0.336
Gender			
Male	18 (41.9%)	21 (46.7%)	0.674
Female	25 (58.1%)	24 (53.3%)	

Values were expressed as n (percentage, %) or median (IQR). p values were derived from Mann-Whitney test. Fisher's exact test was used for assessing distribution of observations or phenomena between different groups.

### Safety and adverse events

No serious adverse events were reported in either group during the 4-week treatment period. In the Observation group, three participants (6.7%) experienced mild, transient scalp redness that resolved spontaneously within 30 min after ozone hydrotherapy. No participants discontinued treatment due to adverse events. In the Control group, no treatment-related adverse events were reported.

### Comparison of clinical efficacy between the two groups after treatment

Clinical efficacy was evaluated after 4 weeks of treatment based on predefined criteria stratifying patient responses into four categories: complete recovery, markedly effective, effective, and ineffective (Table 2). While the distribution of individual response categories did not differ significantly between groups (*p* = 0.531), the overall response rate was significantly higher in the Observation group compared to the Control group (91.1 vs. 69.8%; *p* = 0.015). These findings indicate that the combination of ozone water and scalp complex acid demonstrates enhanced therapeutic efficacy compared to monotherapy.

**Table 2:** Comparison of clinical efficacy between the two groups after the treatment.

Clinical efficacy	Study group		*p*-value
	Control (*n* = 43)	Observation (*n* = 45)	
Recovery	5 (11.6%)	12 (26.7%)	0.531
Effectual	11 (25.6%)	16 (35.5%)	
Effective	14 (32.6%)	13 (28.9%)	
Ineffective	13 (30.2%)	4 (8.9%)	
Total efficacy	30 (69.8%)	41 (91.1%)	0.015

Values were expressed as n (percentage, %). p value was derived from Chi-square test or Fisher's exact.

### Comparison of skin damage scores between groups before and after treatment

To further evaluate treatment efficacy, skin lesion severity was assessed among five domains, including macula, scaling, pruritus, sebum secretion, and lesion area at baseline and after 4 weeks of treatment (Table 3). At baseline, no significant differences were observed between the Control and Observation groups in any parameter (all *p* > 0.05), confirming balanced disease severity before intervention. Post-treatment analysis revealed significantly greater clinical improvement in the Observation group across all domains: macula resolution (*p* = 0.011), reduction in scaling (*p* = 0.028), alleviation of pruritus (*p* = 0.014), decreased sebum production (*p* = 0.004), and a marked reduction in skin lesion area (*p* = 0.009). Notably, improvement in lesion area was also more pronounced in the Observation group, with 35.6% of patients showing complete clearance compared to 11.6% in the Control group (*p* = 0.009). These findings further corroborate the enhanced clinical efficacy of ozone water combined with scalp complex acid in alleviating the multifaceted cutaneous manifestations associated with seborrheic dermatitis.

**Table 3:** Comparisons of skin damage score between the two groups before and after the treatment.

Skin damage score	Before treatment			After treatment		
	Control (*n* = 43)	Observation (*n* = 45)	*p*-value	Control (*n* = 43)	Observation (*n* = 45)	*p*-value
Macula						
0	0 (0.0%)	0 (0.0%)	0.693	7 (16.3%)	15 (33.3%)	0.011
1	9 (20.9%)	10 (22.2%)		15 (34.9%)	18 (40.0%)	
2	20 (46.5%)	17 (37.8%)		15 (34.9%)	11 (24.4%)	
3	14 (32.6%)	18 (40.0%)		6 (13.9%)	1 (2.2%)	
Scale						
0	0 (0.0%)	0 (0.0%)	0.804	6 (14.0%)	14 (31.1%)	0.028
1	13 (30.2%)	14 (31.1%)		18 (41.9%)	19 (42.2%)	
2	22 (51.2%)	20 (44.4%)		14 (32.6%)	10 (22.2%)	
3	8 (18.6%)	11 (24.4%)		5 (11.6%)	2 (4.4%)	
Pruritus						
0	0 (0.0%)	0 (0.0%)	0.738	7 (16.3%)	16 (35.6%)	0.014
1	15 (34.9%)	17 (37.8%)		17 (39.5%)	16 (35.6%)	
2	19 (44.2%)	20 (44.4%)		11 (25.6%)	13 (28.9%)	
3	9 (20.9%)	8 (17.8%)		8 (18.6%)	0 (0.0%)	
Sebum						
0	0 (0.0%)	0 (0.0%)	0.743	5 (11.6%)	14 (31.1%)	0.004
1	15 (34.9%)	17 (37.8%)		13 (30.2%)	17 (37.8%)	
2	18 (41.9%)	19 (42.2%)		17 (39.5%)	11 (24.4%)	
3	10 (23.3%)	9 (20.0%)		8 (18.6%)	3 (6.7%)	
Skin damage area						
0	0 (0.0%)	0 (0.0%)	0.539	5 (11.6%)	16 (35.6%)	0.009
1	11 (25.6%)	14 (31.1%)		17 (39.5%)	15 (33.3%)	
2	19 (44.2%)	20 (44.4%)		16 (37.2%)	13 (28.9%)	
3	13 (30.2%)	11 (24.4%)		5 (11.6%)	1 (2.2%)	

Values were expressed as n (percentage, %). p value was derived from Wilcoxon rank-sum test.

### Changes in total skin damage score, ASFS, and scalp sebum content before and after treatment

**Figure 2:**
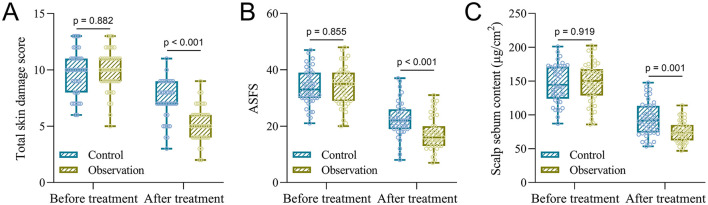
Comparisons of total skin damage score **(A)**, ASFS **(B)**, and scalp sebum content **(C)** between the two groups before and after the treatment. Data were shown with a box plot. *p* values were calculated from the Two-way ANOVA test followed by Tukey's multiple comparisons test.

To further evaluate treatment efficacy, total skin damage score (Figure 2A), ASFS (Figure 2B), and scalp sebum content (Figure 2C) were assessed at baseline and after the 4-week intervention. At baseline, no significant differences were observed between the Control and Observation groups among all parameters: mean total skin damage scores were 9.77 ± 1.95 and 9.93 ± 1.79 (*p* = 0.882); ASFS values were 33.63 ± 6.34 and 34.27 ± 6.82 (*p* = 0.855); and scalp sebum levels were 146.31 ± 28.17 μg/cm^2^ and 148.27 ± 29.83 μg/cm^2^, respectively (*p* = 0.919).

Post-treatment, the Observation group exhibited significantly greater improvements among all measures. The total skin damage score decreased to 5.27 ± 1.51, compared to 7.28 ± 1.71 in the Control group (*p* < 0.001); ASFS declined to 17.42 ± 5.82 vs. 22.37 ± 6.15 (*p* < 0.001); and scalp sebum content was reduced to 74.99 ± 15.39 μg/cm^2^ vs. 94.08 ± 24.88 μg/cm^2^ (*p* = 0.001). These findings further support the better therapeutic efficacy of ozone water combined with scalp complex acid in reducing scaling, seborrhea, and overall lesion severity in patients with SSD.

### Changes in serum IL-1β and TNF-α levels before and after treatment

**Figure 3:**
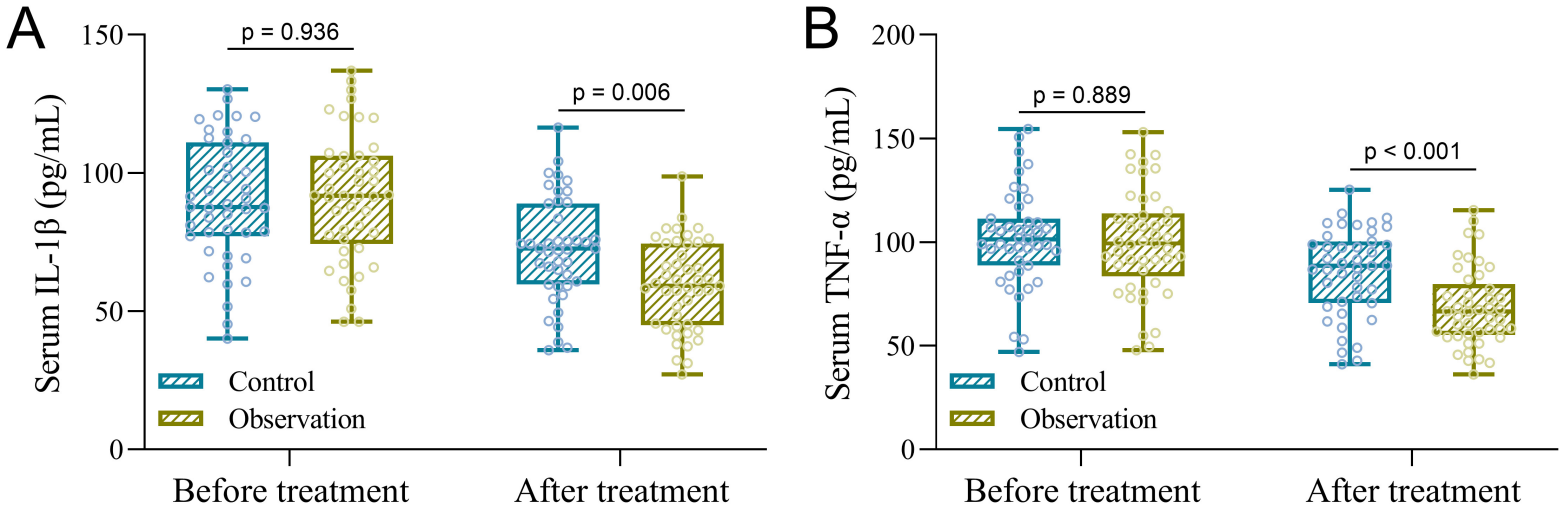
Comparisons of serum IL-1β **(A)** and TNF-α **(B)** between the two groups before and after the treatment. Data were shown with a box plot. *p* values were calculated from the Two-way ANOVA test followed by Tukey's multiple comparisons test.

To evaluate the systemic anti-inflammatory effects of treatment, serum concentrations of IL-1β and TNF-α were measured in both groups before and after the 4-week intervention. At baseline, the mean IL-1β level was 89.79 ± 22.48 pg/mL in the Control group and 91.19 ± 23.32 pg/mL in the Observation group, with no statistically significant difference among them (*p* = 0.936; Figure 3A). Similarly, TNF-α levels were 101.83 ± 23.55 pg/mL in the Control group and 99.79 ± 25.25 pg/mL in the Observation group (*p* = 0.889; Figure 3B), confirming comparable inflammatory status at baseline.

Following treatment, IL-1β concentrations decreased to 72.34 ± 18.75 pg/mL in the Control group and to a significantly lower level of 59.20 ± 16.19 pg/mL in the Observation group (*p* = 0.006, Figure 3A). TNF-α levels showed a similar trend, declining to 85.27 ± 21.06 pg/mL in the Control group and to 68.33 ± 18.95 pg/mL in the Observation group (*p* < 0.001, Figure 3B). These results demonstrate that ozone water combined with scalp complex acid provides an enhanced anti-inflammatory effect in patients with SSD.

## Discussion

This study evaluated the clinical efficacy of ozone hydrotherapy combined with scalp complex acid in patients with moderate to severe SSD. The combination therapy achieved a significantly higher overall response rate than the Control group (91.1 vs. 69.8%, *p* = 0.015) and led to marked improvements in erythema, scaling, and pruritus. Because the study design compared combination therapy against monotherapy, the results support the incremental benefit of adding ozone hydrotherapy but do not permit conclusions regarding the efficacy of ozone hydrotherapy as a standalone treatment. These findings indicate that the combined regimen effectively targets the multifaceted clinical features of SSD.

Previous randomized trials in scalp seborrhoeic dermatitis show that antifungal shampoos (e.g., ciclopirox olamine or ketoconazole) improve key symptoms such as scaling and pruritus over similar treatment periods (22). Consistent with this, our combination regimen was associated with clinically meaningful improvements in symptom scores, sebum measures, and inflammatory biomarkers, although differences in study design and endpoints limit direct comparisons. Systematic reviews of ozone-based dermatologic interventions suggest potential antimicrobial and anti-inflammatory effects, but the evidence remains heterogeneous and follow-up is generally short (17, 23). Accordingly, mechanistic interpretations should be viewed as hypothesis-generating because microbiome and barrier function were not directly assessed.

This study supports the efficacy of a multi-target intervention strategy for moderate to severe SSD. The combined therapy, consisting of complex acid and ozone water, led to significant improvements in key symptoms. The Observation group showed greater reductions in scale and ASFS scores, highlighting the potential complementary effects of keratinolysis and anti-inflammatory effects in correcting epidermal differentiation abnormalities (6, 12). Sebum secretion in the Observation group was significantly reduced, which may be associated with changes in sebaceous activity or inflammation; however, the specific metabolic targets were not directly assessed in this study (24, 25). Furthermore, pruritus relief was notable, indicating the combined therapy's potential benefit on pruritus-related pathways (26, 27). Lesion scores improved significantly across five core indicators, including erythema, scaling, pruritus, sebum secretion, and lesion area. The complete lesion clearance rate was 35.6% in the Observation group compared to 11.6% in the Control group, with a 47.0% reduction in total skin damage score. These results suggest that the regimen may improve clinical manifestations through combined keratolytic, antimicrobial, and anti-inflammatory effects; however, Malassezia colonization and skin barrier function were not directly measured in this study (6, 12).

The therapy may influence the scalp microenvironment, as indicated by a strong correlation between ASFS and sebum content reduction. Complex acid reduces scale adhesion by modulating keratinocyte differentiation (28), while ozone water's antimicrobial effect may help interrupt the feedback loop between sebaceous breakdown and inflammation (6, 29); however, microbiome changes were not assessed. These microenvironmental changes may explain the more sustained relief observed in the Observation group (30). In conclusion, this study provides clinical evidence for a multi-target intervention strategy in seborrheic dermatitis treatment. The synergistic actions of complex acid and ozone water offer a promising therapeutic approach, particularly for patients with severe scaling and seborrhea. Further studies should explore the underlying molecular mechanisms, particularly through skin lipidomics.

Ozone hydrotherapy may reduce IL-1β and TNF-α levels through a multi-target regulatory mechanism. First, ozone has been reported to inhibit the NF-κB signaling pathway by downregulating IκBα phosphorylation and preventing NF-κB p65 nuclear translocation, thus blocking the transcription of pro-inflammatory factors (31). Studies show that ozone reduces NF-κB protein expression and suppresses the production of downstream inflammatory mediators, such as iNOS and COX-2 (18), which is consistent with the greater reduction of IL-1β/TNF-α in the Observation group, although these signaling events were not directly measured in our participants. Additionally, ozone may regulate the oxidative stress-inflammation axis by increasing antioxidant enzyme activity (SOD, CAT, and GSH) (18) and reducing lipid peroxidation products, such as MDA (32, 33), thereby disrupting the oxidative stress-driven NF-κB activation loop. This dual mechanism may underlie the systemic anti-inflammatory effects of ozone, as the crosstalk between oxidative stress and NF-κB signaling constitutes a fundamental axis in the regulation of cutaneous inflammatory responses. Lastly, ozone may help rebalance the cytokine network by inhibiting pro-inflammatory cytokines (IL-6, IFN-γ) and upregulating anti-inflammatory IL-10 (34), leading to a Th1/Th2 immune shift. Specifically, ozone oxidative preconditioning can suppress the TLR4/ NF-κB signaling cascade (35), further reduce IL-1β/TNF-α release, aligning with findings that ozone regulates the Nrf2/HO-1 antioxidant axis to inhibit inflammasome activation (36). The superior therapeutic efficacy of ozone water–based combination therapy in seborrheic dermatitis may be attributed to a triadic mechanism involving activation of endogenous antioxidant enzymes, inhibition of NF-κB signaling, and modulation of proinflammatory cytokine expression; however, these mechanistic interpretations should be considered hypothesis-generating because these pathways were not directly tested in this clinical trial.

The combined use of ozone hydrotherapy and scalp complex acid offers a compelling therapeutic strategy for the management of seborrheic dermatitis. This approach may provide multifaceted clinical benefits by simultaneously targeting microbial factors and inflammatory dysregulation. Notably, ozone hydrotherapy demonstrates a favorable safety profile, as evidenced by both preclinical data and findings from the present study, and poses a lower risk of adverse effects relative to long-term corticosteroid or antifungal use. Moreover, the concurrent reduction in local and systemic inflammatory markers suggests potential for sustained disease control, although durability of response and relapse rates were not assessed in this 4-week study.

Although the findings of this study are promising, there are a few limitations that should be noted. First, the sample size was moderate, and the study was conducted at a single center, which may limit how widely the results can be applied. To better confirm the efficacy and safety of this combination therapy, a larger, multi-center trial is needed. Second, while significant improvements in clinical symptoms and inflammatory markers were observed, the study did not directly measure changes in the skin microbiome or barrier function, which could provide further insights into the underlying mechanisms. Third, long-term relapse was not assessed beyond the 4-week treatment period. In addition, the between-group imbalance in treatment frequency may have introduced performance bias and contributed to the observed differences. Future trials should standardize session frequency and contact time to better isolate the treatment effect of ozone hydrotherapy. Fourth, the primary analysis was conducted on a per-protocol basis, as participants who withdrew during the intervention were excluded. An intention-to-treat analysis was not performed, which may introduce attrition bias and limit the generalizability of the findings. Future studies should adopt intention-to-treat principles to better preserve the benefits of randomization.

## Conclusion

This study shows that combining ozone hydrotherapy with scalp complex acid significantly improves clinical outcomes in patients with moderate to severe SSD. The combined treatment led to a marked reduction in lesion severity, scalp sebum production, and inflammatory markers such as IL-1β and TNF-α. However, the study design does not allow conclusions about the efficacy of ozone hydrotherapy alone. These results suggest that the combination approach may offer incremental benefits over scalp complex acid monotherapy in the management of SSD.

## Data Availability

The original contributions presented in the study are included in the article/supplementary material, further inquiries can be directed to the corresponding author.
